# Reliability of AI-Automated and Semiautomated Upper Airway Volume Segmentation

**DOI:** 10.3390/diagnostics16071105

**Published:** 2026-04-07

**Authors:** Natalia Chwarścianek, Natalia Kazimierczak, Zbigniew Serafin, Wojciech Kazimierczak

**Affiliations:** 1 Independent Researcher, 85-796 Bydgoszcz, Poland; 2 Kazimierczak Clinic, Dworcowa 13/u6a, 85-009 Bydgoszcz, Poland; natnowicka@gmail.com (N.K.); w.kazimierczak@cm.umk.pl (W.K.); 3 Faculty of Medicine, Bydgoszcz University of Science and Technology, Kaliskiego 7, 85-796 Bydgoszcz, Poland; zbigniew.serafin@pbs.edu.pl; 4 Department of Radiology and Diagnostic Imaging, Collegium Medicum, Nicolaus Copernicus University in Torun, Jagiellońska 13-15, 85-067 Bydgoszcz, Poland

**Keywords:** oral radiology, digital dentistry, dental imaging, diagnosis, treatment planning

## Abstract

**Background/Objective**: To evaluate the reliability, diagnostic accuracy and time efficiency of an artificial intelligence (AI)-automated method (CephX) and a semiautomated method (INVIVO) for upper airway segmentation, the manual digital method (ITK-SNAP) was used as the reference standard. **Methods**: This retrospective study analyzed cone-beam computed tomography (CBCT) scans from 133 patients. The upper airway volume and narrowest cross-sectional area were measured via the three methods. Reliability and repeatability were assessed via the intraclass correlation coefficient (ICC). The time required for each segmentation method was also recorded and compared. **Results**: Both the AI-automated (ICC = 0.945) and semiautomated (ICC = 0.992) methods demonstrated excellent reliability for total volume measurements compared with the manual reference. For the narrowest area, the automated method showed excellent agreement (ICC = 0.943), whereas the semiautomated method showed good agreement (ICC = 0.868). All methods demonstrated excellent intrareader repeatability (ICC > 0.95) and high test–retest reliability. The AI-automated method was significantly more time-efficient, requiring less than 30 s per analysis, compared with 161.4 s for the semiautomated method and 336.6 s for the manual method. **Conclusions**: AI-automated and semiautomated segmentation methods are reliable and accurate alternatives to manual upper airway analysis. The AI-based approach offers a substantial advantage in time efficiency, making it a valuable tool for clinical practice.

## 1. Introduction

The upper airways consist of the nasal cavities, pharynx and larynx. The pharynx, the region most prone to collapse, is divided into 3 parts: the nasopharynx, oropharynx, and hypopharynx. The nasopharynx begins in the choanae (posterior opening of the nasal cavities) and ends on the hard palate. The oropharynx ranges from the uvula to the epiglottis. The hypopharynx spans the area from the epiglottis to the vocal cords, where the trachea begins [1]. Functions of the upper airway include air warming and humidification, pathways for olfaction, coordination of ventilation with swallowing and protection from aspiration of food, primary defense against infection, and especially for humans, speech [2].

Traditionally, airway dimensions are assessed via lateral cephalograms [3]. However, cephalometric measurements have severe limitations in assessing the airway, as only changes in the sagittal and vertical dimensions can be observed [4]. Three-dimensional (3D) computed tomography (CT) and cone-beam CT (CBCT) are more promising techniques than lateral cephalograms for upper airway assessment [5,6]. With the advent of CBCT imaging, our understanding of airway morphology has been expanded to 3 dimensions to include the overall volume and, perhaps most physiologically relevant, the cross-sectional area perpendicular to the direction of air flow, as visualized in the axial plane [7].

Volumetric analysis of the upper airway can aid in identifying conditions such as obstructive sleep apnea syndrome (OSAS), craniofacial abnormalities, and orthodontic or dentofacial deformities [3,8,9,10]. The segmentation process for CBCT records enables detailed monitoring of airway boundaries by removing surrounding anatomical structures. Segmentation can be performed via three different methods: manual, semiautomatic, and automatic [11]. Manual segmentation, where an expert meticulously traces the airway slice-by-slice, is widely considered the gold standard reference method [8,12]. However, this process is time-consuming, tedious and expert-dependent, which negatively affects efficiency and accuracy and can lead to missed diagnoses of the disease; all of the above can have significant impacts on human health [5,13].

Recently, artificial intelligence (AI) and deep learning techniques, which employ computers or machines to imitate human logic and cognition to complete a series of intelligent tasks, have seen extensive use in medical imaging [14]. Several studies have assessed the diagnostic performance of artificial intelligence integrated with CBCT imaging in relation to oral and maxillofacial anatomical landmarks and lesions [10,15,16]. However, researchers have reported differing values of accuracy [17]. In recent years, there has been a marked increase in the number of commercially available software solutions that utilize artificial intelligence. Examples of such AI platforms include CephX (CephX, Las Vegas, NV, USA) and Invivo (Anatomage, San Jose, CA, USA), both of which provide automated analyses of CBCT examinations, including cephalometric analyses and assessments of airway volume. However, their reliability—and consequently patient safety—is often untested or has, in certain applications, been recognized as unsatisfactory [18,19]. To the best of our knowledge, no previous study has evaluated the accuracy and efficacy of these two programs specifically for volumetric assessment of the upper airways.

Although multiple studies have investigated upper airway segmentation using CBCT, much of the existing literature focuses on research prototypes or on software platforms that differ from tools used routinely in dental and orthodontic workflows [20,21,22]. In contrast, the present study provides an independent, clinically oriented validation of two commercially available and commonly used solutions—one fully AI-automated (CephX) and one semiautomated (Invivo 7)—against a manual digital reference standard (ITK-SNAP). Beyond agreement in total volume, we also evaluate the narrowest cross-sectional area, quantify time-efficiency, and document automated-processing failures observed in routine clinical CBCT data, which together address practical questions relevant to implementation and patient safety.

The primary purpose of this study was to compare three methods of measuring upper airway volumes and the duration of these measurements. Automatically using the AI—based program CephX, semiautomatically using the Invivo 7 program, and manually—digitally using the ITK SNAP program. This allowed us to verify whether the semi-automatic and automatic methods are as good as the reference method (manual measurement).

## 2. Materials and Methods

### 2.1. Study Design

This retrospective study aimed to evaluate the reliability of AI-automated and semiautomated methods for upper airway volume segmentation in comparison to manual digital segmentation, which is considered the gold standard. The study was conducted in accordance with the Declaration of Helsinki and approved by the Ethics Committee of Collegium Medicum, Nicolaus Copernicus University in Torun, Poland (protocol No. 274/2025, 23 April 2025). This study was conducted retrospectively and was therefore not formally registered in a public trials registry. As this was a retrospective study utilizing existing imaging data, there were no adverse events related to the performance of the index tests or the reference standard. The full study protocol is available from the corresponding author upon reasonable request.

### 2.2. Participants

Our study was conducted on 165 consecutive patients referred for large field-of-view (FOV) CBCT scans at a private dental center between January 2024 and March 2025. The main indications for CBCT scans were impacted teeth and suspicion of periapical lesions on both sides of the dental arch. The inclusion criteria were high-quality CBCT scans of the maxillofacial region with complete visualization of the upper airways, no history of prior surgical interventions in the upper airway region, and no visible artifacts affecting segmentation. The exclusion criteria included scans with significant artifacts, incomplete visualization of the upper airways, or prior surgeries altering upper airway anatomy. After the initial selection of patients with CBCT images, 27 patients were excluded because the scan region did not cover the upper airways. Finally, 138 patients were included in the study group.

### 2.3. CBCT Image Acquisition

CBCT scans were acquired using a Hyperion X9 PRO (MyRay, Imola, Italy) machine. One standard, marked as the “Regular” setting of the apparatus, was used (90 kV, 36 mAs, CTDI/Vol 4.09 mGy, and 13 cm FOV). All the CBCT images were reconstructed with a slice thickness of 0.3 mm. All the scans were stored in Digital Imaging and Communications in Medicine (DICOM) format.

### 2.4. Upper Airway Definition

In this study, the “upper airway” refers to the pharyngeal airway lumen (air space) segmented within standardized anatomical limits in CBCT [8,12]. The segmented region was defined using two boundary planes identified on the mid-sagittal reconstruction:

- Superior boundary plane (Plane S): a plane parallel to the palatal plane passing through the posterior nasal spine (PNS).
- Inferior boundary plane (Plane I): a plane parallel to the palatal plane passing through the most superior tip of the epiglottis (E).

**Figure 1:**
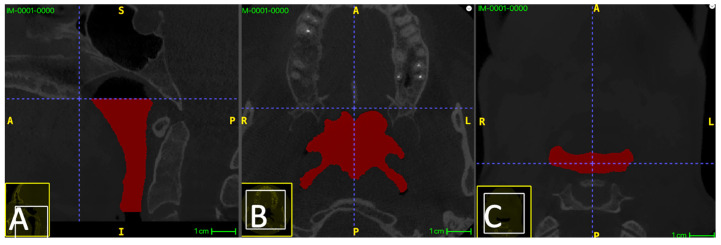
Definition of the segmented upper airway region. (**A**) Mid-sagittal CBCT view showing the superior boundary plane (Plane S) passing through posterior nasal spine (PNS) and parallel to the palatal plane, and the inferior boundary plane (Plane I) passing through the superior tip of the epiglottis and parallel to the palatal plane. The segmented pharyngeal airway lumen is highlighted in red. (**B**) Axial slice at the superior boundary (Plane S). (**C**) Axial slice at the inferior boundary (Plane I).

The airway lumen was segmented between Plane S and Plane I, bounded laterally and posteriorly by the pharyngeal soft-tissue walls and anteriorly by the posterior contour of the soft palate and tongue base (air–soft tissue interface). Air spaces outside the defined pharyngeal region (e.g., external air) were excluded by restricting segmentation to the ROI between Plane S and Plane I and by using connectivity-based region selection when applicable. The adopted boundaries are illustrated in Figure 1.

### 2.5. Upper Airway Segmentation

Three distinct methods were employed to segment the upper airway volume:

1. AI-Automated Segmentation: CephX 4.02 (CephX, Las Vegas, NV, USA) software was used for automated segmentation of the upper airway volume. This AI-powered tool automatically delineates the airway boundaries on the basis of predefined algorithms.
2. Semiautomated segmentation: Invivo 7 software (Anatomage, San Jose, CA, USA; Invivo Desktop version 7.2.6) was used for semiautomated segmentation. After importing DICOM data, the operator defined the superior and inferior limits using the two planes (Plane S at PNS and Plane I at epiglottis tip), both parallel to the palatal plane, thereby creating a standardized ROI. A threshold/region-based airway segmentation tool was then applied within this ROI to select air voxels (air-density region) and generate an initial airway mask. The mask was inspected in sagittal, axial, and coronal views, and manual corrections were performed when needed to remove leakage into adjacent non-target air spaces and to ensure that only the pharyngeal airway lumen within the ROI was included. After final approval of the segmentation, Invivo7 automatically calculated total airway volume (cm^3^) and narrowest cross-sectional area (mm^2^ or cm^2^, as reported by the software).
3. Manual Digital Segmentation: ITK-SNAP (Penn Image Computing and Science Laboratory, Philadelphia, PA, USA, version 4.2.2) was used for manual digital segmentation. This open-source software allows for precise, slice-by-slice delineation of the upper airway boundaries by trained operators. After DICOM import, the operator defined the same superior and inferior limits (Plane S at PNS; Plane I at epiglottis tip; both parallel to the palatal plane). The airway lumen was then delineated slice-by-slice within these limits. Segmentation was performed primarily in the sagittal plane with continuous verification in axial and coronal views. The airway boundary was traced along the air–soft tissue interface using polygon/brush tools; when connectivity tools were used, only the connected pharyngeal airway lumen within the ROI was retained to prevent inclusion of external air. The software-derived 3D label was used to obtain total airway volume. The narrowest cross-sectional area for the manual method was determined by reviewing all axial slices within the ROI and recording the minimal lumen area.

The results of the segmentation processes were manually entered into spreadsheets.

### 2.6. Manual Segmentation Protocol

All segmentations were performed independently by 2 operators with expertise in CBCT image analysis. To minimize bias, the operators were blinded to the segmentation results from the other methods. Before finalizing each segmentation, the operator verified the superior and inferior boundary planes (Plane S at PNS; Plane I at epiglottis tip) and visually inspected the segmentation in three orthogonal planes to ensure (I) confinement to the ROI, (II) exclusion of non-target air spaces, and (III) anatomically plausible airway boundaries. A subset of cases (20%) was reanalyzed by Reader 1 to assess the intra-reader agreement.

### 2.7. Time Measurement

The time taken to complete each segmentation and volume and narrowest area measurement was recorded in seconds.

### 2.8. Sample Size

The sample size calculation was performed using the Wald method, considering the expected intraclass correlation coefficient (ICC) values from 0.80–0.95 on the basis of preliminary data [23]. Precision levels of ±0.025 and ±0.05 were used to estimate the required sample sizes. The anticipated ICC values indicated the need for sample sizes ranging from 11 (ICC = 0.95; ±0.05) to 555 (ICC = 0.80; ±0.025). Given our final sample of 133 patients, the study is adequately powered to detect ICC values of ≥0.90 with ±0.05 precision.

### 2.9. Statistical Analysis

The means, standard deviations, medians, quartiles, and ranges of the quantitative variables were calculated. The reliability of the quantitative measures was assessed with an intraclass correlation coefficient of type 2 or 3 (according to Shrout and Fleiss), as appropriate. Spearman’s correlation coefficient was used to assess the correlation between two quantitative variables. The sample size was calculated using the Wald method [23]. The significance level was set to 0.05. All the analyses were conducted in R software, version 4.5.1.

## 3. Results

### 3.1. Study Population

In this retrospective study, the initial population comprised 138 patient CBCT scans. Five patients were subsequently excluded because their scans lacked CephX reports; the AI algorithm was unable to process these datasets. Although those datasets were successfully processed via Invivo software and manually via ITK-SNAP, they were nonetheless excluded from the final analysis. Consequently, the study population was reduced to 133 patients. The mean age of the participants was 26.7 ± 10.04 years, with a median age of 26 years and an overall range of 8–54 years. Among the 133 participants, 87 (65.41%) were female, and 46 (34.69%) were male. The demographic characteristics of the patients in the final study group are presented in Table 1 and Figure 1.

### 3.2. Volume and Area Measurements

**Figure 2:**
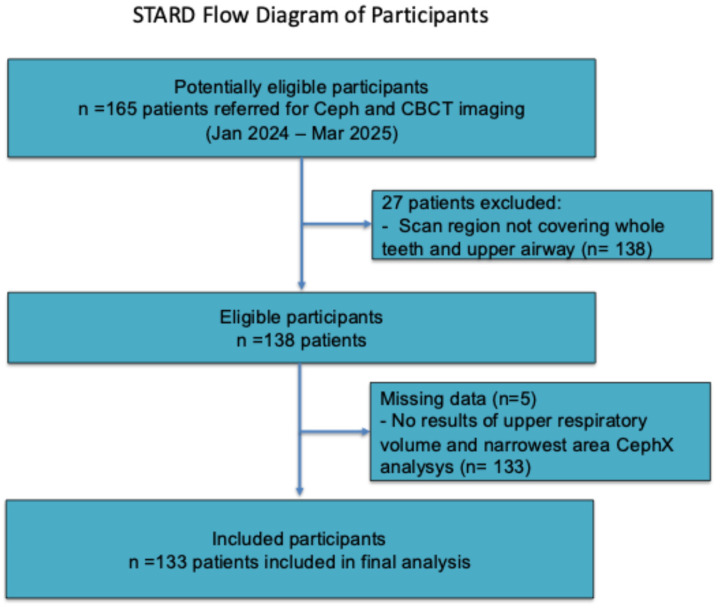
STARD participant flow diagram for study participants.

Detailed descriptive statistics for total airway volume were calculated for each measurement method and are presented in Table 2. For total airway volume, the mean volume was highest with the CephX method for the male sex (mean = 18.41 cm^3^, SD = 10.71) and lowest with the INVIVO method for the female sex (mean = 15.76 cm^3^, SD = 6.59). The reference method, ITK SNAP, yielded a mean volume of 16.55 cm^3^ (SD = 7.66) for Reader 1 and 16.77 cm^3^ (SD = 7.89) for Reader 2. The mean airway volume was greater in males than in females for all three methods and for both Readers. Figure 2 presents the case with the greatest differences among all three methods of volume measurement.

**Figure 3:**
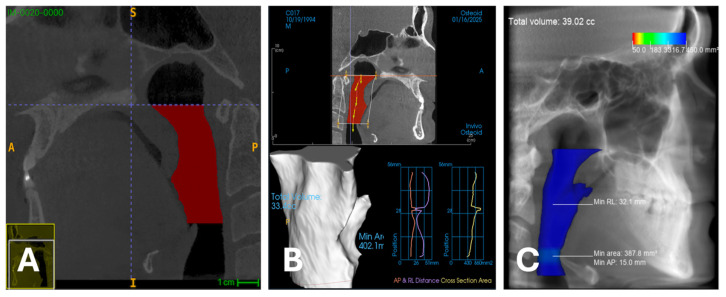
The samples with the greatest differences between manual and semiautomatic and automatic volume measurements were used. (**A**)—manual segmentation, volume 30.7 cm^3^; (**B**)—semiautomatic segmentation; volume 33.4 cm^3^; (**C**)—automatic segmentation, volume 39.0 cm^3^.

Detailed descriptive statistics for the narrowest airway area were calculated for each measurement method and are presented in Table 3. For the narrowest area measurements, the mean area was highest with the CephX method for the male sex (mean = 218.54 cm^2^, SD = 107.31) and lowest with the INVIVO method for the female sex (mean = 176.21 cm^2^, SD = 97.24). The reference method, ITK SNAP, yielded a mean area of 208.03 cm^2^ (SD = 109.8) for Reader 1 and 205.31 cm^2^ (SD = 105.86) for Reader 2. Figure 3 presents the case with the greatest differences among all three methods of area measurement.

### 3.3. Reliability of the Automated and Semiautomated Methods Compared with the Reference Method

The reliability of the automated and semiautomated measurements compared with that of the reference method (the mean results of both Readers manual segmentation via ITK SNAP) was evaluated via the ICC3 (Table 4). Both CephX (ICC = 0.945; 95% CI: 0.923–0.961) and INVIVO (ICC = 0.992; 95% CI: 0.989–0.994) showed excellent agreement for total volume measurements. For the narrowest area, CephX exhibited excellent agreement (ICC = 0.943; 95% CI: 0.920–0.959), whereas INVIVO had lower but still good agreement (ICC = 0.868; 95% CI: 0.819–0.905).

### 3.4. Correlation Between Total Volume and Narrowest Area

A strong positive correlation was found between the total airway volume and the narrowest area, as measured by the ITK SNAP (Table 5). The Spearman correlation coefficient was 0.858, with a *p* value of less than 0.001, indicating statistical significance. This suggests that a larger total airway volume is associated with a larger narrowest area. Figure 2 illustrates this relationship.

### 3.5. Repeatability of Automated, Semiautomated, and Manual Measurements and Intrareader Agreement

Repeatability was assessed via the intraclass correlation coefficient (ICC2), which is based on test–retest measurements conducted on 20% of the study group by Reader 1. All methods demonstrated excellent repeatability for total volume measurements: CephX (ICC = 0.998; 95% CI: 0.997–0.999), ITK SNAP (ICC = 0.992; 95% CI: 0.989–0.995), and INVIVO (ICC = 0.995; 95% CI: 0.992–0.996). For the narrowest airway area measurements, CephX (ICC = 0.997; 95% CI: 0.996–0.998) and ITK SNAP (ICC = 0.957; 95% CI: 0.941–0.970) showed excellent repeatability, whereas INVIVO demonstrated good repeatability (ICC = 0.832; 95% CI: 0.738–0.890). The intrareader agreement (ICC2) demonstrated excellent consistency for the ITK SNAP measurements for the total volume (ICC = 0.99; 95% CI: 0.985–0.993) and the narrowest area (ICC = 0.95; 95% CI: 0.931–0.964). The detailed results of the repeatability calculations are presented in Table 6.

The interreader repeatability of manual measurements via ITK-SNAP software (Penn Image Computing and Science Laboratory, Philadelphia, PA, USA, version 4.2.2) demonstrated excellent agreement for both volume and area assessments, with intraclass correlation coefficients of 0.952 and 0.944, respectively.

### 3.6. Time-Efficiency Methods

Manual measurements required an average of 207.6 s per CBCT dataset, whereas semiautomatic measurements required an average of 161.4 s per test. In contrast, for measurements performed by artificial intelligence, it was sufficient to load the CBCT scan into the program and retrieve the analysis, keeping the image transfer time to less than 30 s per case; each analysis became available within 15 min of submission.

Using the reference method, the average time to measure the narrowest airway region was 129 s. The results of the narrowest area calculations performed by Invio and CephX software were delivered automatically alongside the volume calculations. Overall, manual volume measurements and manual measurements of the narrowest area took on average 336.6 s (Table 7).

### 3.7. Sample Size Calculations

Post hoc analysis of the sample size was conducted on the basis of the obtained ICC values. For ICC values between 0.80 and 0.95, the required sample sizes for precision ±0.05 ranged from 11–139. Thus, the final sample size of 133 participants provides sufficient statistical power for ICC estimates ≥0.85 at a precision of ±0.05, indicating adequate power for assessing the reliability and repeatability of the segmentation methods used in this study.

## 4. Discussion

The primary aim of this study was to evaluate the reliability and time efficiency of an AI-automated (CephX) and a semiautomated (INVIVO) method for upper airway segmentation against the manual digital approach (ITK-SNAP), which is considered the gold standard. Our findings demonstrate that both automated and semiautomated methods offer excellent reliability for measuring total airway volume compared with the manual reference. For the narrowest airway area, the automated method also showed excellent agreement, whereas the semiautomated method showed good agreement. Furthermore, all three methods exhibited high intrareader repeatability, underscoring their consistency. The most significant advantage of the AI-automated method was its profound time efficiency, reducing the analysis time from over five minutes for the manual method to less than 30 s.

AI stands as transformative power in today’s digital revolution, affecting various economic sectors by performing tasks that typically require human intelligence. Its introduction to dentistry is particularly notable, offering new and improved ways to enhance diagnostic imaging, plan treatments, and manage patient care [24]. AI can perform tasks with greater precision and accuracy than humans [25]. Moreover, AI can be continually trained and refined with extensive datasets, leading to increasingly accurate and reliable outcomes [26]. As the integration of AI within dentistry continues to advance, it faces significant technical challenges that could impede its efficacy. However, integrating AI into clinical practice is accompanied by challenges, especially regarding the availability and quality of training datasets, which are critical for ensuring system accuracy [27].

A key contribution of this work is that it evaluates commercial tools as deployed in routine clinical workflows, rather than a research-only algorithm under controlled conditions and available only for research teams. By integrating assessments of both volume and the narrowest area, alongside direct comparisons of workflow time and documentation of failures, this research provides a comprehensive evaluation of the efficacy of readily implementable AI tools in the real-world contexts of dental practices.

CBCT-based measurements of upper airway volume play a significant role in the diagnosis and evaluation of a wide range of diseases and disorders, especially within the fields of otolaryngology, orthodontics, and maxillofacial surgery. The application of AI in dental imaging also involves airway detection and volumetric measurements. Sin et al. [28] evaluated a convolutional neural network (CNN) for the automatic segmentation of pharyngeal airway volumes on the basis of 306 CBCT images and achieved high performance. Similar results were reported by Cho et al. [29], who also used a CNN-based model for the segmentation of these structures and confirmed its effectiveness. Despite these advancements, our study noted slightly lower precision with semiautomatic and automatic methods than with manual segmentation, particularly with respect to anatomical variability. Nevertheless, AI-driven platforms such as CephX offer rapid, reliable evaluations, substantially reducing the interoperator variability inherent to manual methods, and thus serve as valuable adjunctive tools rather than replacements for clinical expertise [30].

Obstructive sleep apnea syndrome (OSAS) is a critical condition linked with airway dimensions. OSAS involves repeated episodes of partial or complete airway obstruction during sleep, significantly impacting patient health [31]. OSA is defined as repeated episodes, greater than 5 per hour, of partial or total obstruction of the upper airways during sleep, leading to airway obstruction (apnea) or reduced airflow (hypopnea) [1]. An apnea event, by definition, should last at least 10 s and is usually associated with sleep or microarousal fragmentation. Hypopnea can be defined as a reduction in ventilation (at least 50%) with an oxygen desaturation of ≥4% [1]. OSA is a very common condition with significant adverse consequences. A narrow, floppy upper airway provides the pathophysiological basis for OSAS. The pharynx tends to collapse at inspiration due to Bernoulli’s effect, which results in partial or complete obstruction [32]. The prevalence and health effects of OSAS are currently receiving increasing attention from dental professionals because of the multidisciplinary nature of the treatment options [33].

Previous research has demonstrated a relationship between the upper airway and stomatognathic development [4]. The volume of the upper airway is a critical factor in orthodontics because it is associated with craniofacial growth and development; it can be influenced by jaw positioning and, in turn, can dictate various treatment plans [34]. When the upper airway is restricted or obstructed, breathing patterns may change, directly affecting normal craniofacial development and dental positioning [35,36,37]. Optimal upper airway function depends on nasal breathing, and for many years, researchers have examined the impact of impaired nasal breathing on craniofacial development and dentition [17].

The primary aim of this study was to assess the reliability of automated and semiautomated segmentation methods—using artificial intelligence—compared with the commonly employed manual segmentation technique for measuring upper airway volumes. A review of the literature revealed no consensus or standardized methodology for measuring the upper airway. However, recent software developments have facilitated rapid, automated analyses of upper airway volume that are accurate, reproducible, and practical. The simplicity and speed of these methods enable widespread adoption without requiring specialized expertise [1]. A notable benefit of both examined software programs is their user-friendliness. In the case of CephX, once the CBCT scan was uploaded, the program produced a complete analysis without further user intervention. Similarly, in vivo, after the relevant anatomical region—with only a basic understanding of respiratory anatomy—is defined, the system automatically generates the necessary measurements.

**Figure 4:**
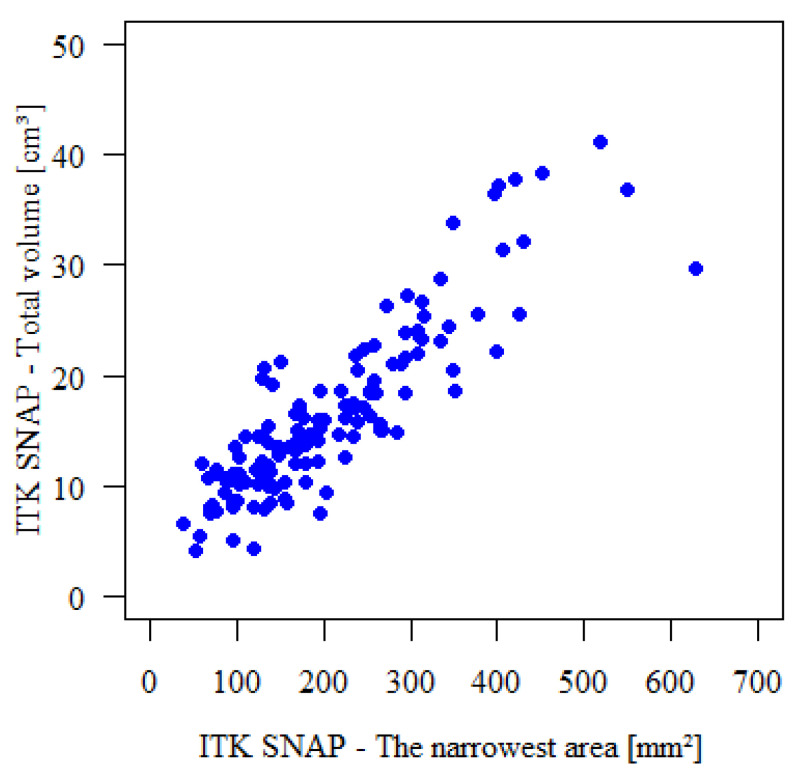
Relationship between total airway volume and narrowest area.

Previous imaging studies in OSA patients using optical coherence tomography during wakefulness have shown that the retropalatal airway is narrower than the retroglossal airway is [38]. Many studies have indicated that patients with OSAS have a narrowed cross-sectional area [23]. Axial CBCT studies typically identify the smallest airway slice at the retropalatal level [39]. The assessment protocol for CBCT described herein constitutes a valuable screening tool for OSAS, which allows clinicians to refer patients to the hospital for diagnostic confirmation [1]. Therefore, measuring the respiratory volume of the upper airways, supplemented with information about the narrowest point in the airways, can be an important element in diagnosing the severity of the disease (Figure 4). The parameter was always calculated via both CephX (AI-based) and Invivo 7 software. When the statistical data were analyzed, the results were almost identical to the manual measurements performed by both readers. This is a major advantage of both programs because, to identify and measure it manually, it is necessary to analyze the entire length of the airways and compare the surface areas of several places, selecting the narrowest one. Manual volumetry is both time-consuming and prone to errors. The time efficiency of AI-automated analysis offers a notable advantage, as the average duration required for manual volumetry―approximately 336.6 s―is nearly eleven times longer than that for AI-automated methods, which typically require fewer than 30 s.

**Figure 5:**
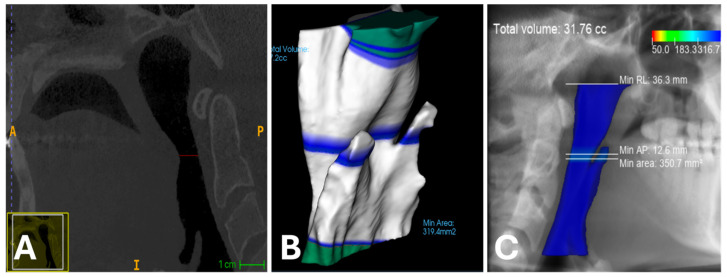
Sample with the greatest differences between manual and semiautomatic and automatic minimal upper airway area measurements. (**A**)—Manual segmentation, 297.3 cm^2^; (**B**)—semiautomatic segmentation, 319.4 cm^2^; (**C**)—automatic segmentation, 350.7 cm^2^.

During the research conducted in this study, a technical issue prevented the CephX software from completing the segmentation process in a few cases. Consequently, 5 CBCT examinations were excluded from the study because the AI software incorrectly indicated an incomplete range of the upper respiratory tract. However, when both ITK SNAP and Invivo 7 were used, measurements could be performed without any problems. This is probably due to the continuous learning of the AI model, which needs a continuous flow of new data to ensure that its results are as close as possible to those of humans [40]. The varied anatomical structure of the airways, including their shape, length, and cross-section, may have contributed to difficulties in precisely determining anatomical points, calculating volumes, and locating the narrowest point, which in turn may have led to discrepancies in the measurements obtained (Figure 3 and Figure 5). However, many articles have demonstrated the potential of artificial intelligence, which includes the ability to assist in diagnosis, suggest appropriate interventions, and, in particular, improve image analysis [40,41,42,43]. As an example of an AI-based system, the CephX program is a useful tool for analyzing the upper respiratory tract, offering significant time savings and high repeatability of measurements; however, in the case of anatomical variability, deviations that require verification by a specialist may exist [44]. However, the recent literature shows highly promising results and encourages further research on the development of AI tools [31].

Despite the demonstrated effectiveness of AI-based segmentation, several limitations should be acknowledged. The retrospective and single-center design may limit generalizability and introduce potential selection bias. Patient populations from a single dental center may not fully represent broader demographic or clinical variability. Additionally, the CBCT datasets were obtained for routine dental indications, and comprehensive medical histories were not consistently available. Therefore, systemic comorbidities potentially affecting upper airway morphology, such as obstructive sleep apnea, were not included in the analysis. Patients were also not stratified according to skeletal pattern or dental class, as the primary aim of this study was methodological validation rather than morphological comparison between subgroups. Future prospective studies incorporating detailed clinical data and skeletal classifications are warranted to further validate the clinical applicability of AI-based airway segmentation.

Despite these limitations, the current study provides important initial validation of AI-driven airway segmentation methods, establishing a foundation upon which future research can be conducted. Ongoing research, particularly prospective, multicenter studies incorporating broader patient demographics and direct clinical outcome measures, will be instrumental in fully evaluating the potential and limitations of AI integration into routine clinical practice.

## 5. Conclusions

In conclusion, AI-automated and semiautomated software provide reliable and accurate segmentation of the upper airways, with a significant advantage in time efficiency compared with manual methods. The CephX platform proved to be an effective tool for rapid airway analysis.

The clinical applicability of CBCT-based airway assessment should, however, be interpreted within appropriate boundaries. Its use appears particularly justified in dental disciplines, including orthodontics and craniofacial evaluation, as well as in the assessment of patients with OSAS. Nevertheless, CBCT should not be considered a primary imaging modality for the diagnosis or treatment planning of diseases involving the larynx and upper trachea.

Although AI-driven technologies show considerable potential for improving efficiency in dental imaging, they should be regarded as adjunctive tools that support rather than replace clinical judgment. This is the first study to comprehensively evaluate and compare the reliability and efficiency of AI-automated and semiautomated upper airway segmentation methods against the manual gold standard on a large sample of CBCT scans. The AI-based approach offers a dramatic improvement in time efficiency, completing measurements in under 30 s versus more than 5 min manually, supporting its clinical applicability.

## Figures and Tables

**Table 1:** Characteristics of the study group.

Parameter		Total (*n* = 133)
Age [years]	Mean (SD)	26.7 (10.04)
	Median (quartiles)	26 (20–34)
	Range	8–54
	*n*	133
Sex	Female	87 (65.41%)
	Male	46 (34.59%)

**Table 2:** Summary of airway volume measurements.

Parameter	Sex	*n*	Mean	SD	Median	Min	Max	Q1	Q3
CephX	Female	87	15.65	7.52	14.1	3.3	40.3	10.28	18.83
	Male	46	18.41	10.71	16.25	2.8	41.8	10.1	23.48
	Total	133	16.6	8.8	14.45	2.8	41.8	10.2	20.2
INVIVO	Female	87	15.76	6.59	14.7	4.1	40	11.25	19.85
	Male	46	17.56	8.73	15.8	5.1	38.9	11.12	21.88
	Total	133	16.38	7.42	14.9	4.1	40	11.2	20.3
ITK SNAP—Reader 1	Female	87	15.91	6.84	14.8	4.2	41.2	10.95	19.35
	Male	46	17.76	8.97	15.45	5.1	38.3	11.22	22.7
	Total	133	16.55	7.66	15	4.2	41.2	11.1	20.6

SD—standard deviation, Q1—lower quartile, Q3—upper quartile.

**Table 3:** Summary of narrowest airway area measurements.

Method	Sex	*n*	Mean	SD	Median	Min	Max	Q1	Q3
CephX	Female	87	218.54	107.31	200.3	12.2	637.7	144.48	282.85
	Male	46	215.77	121.17	179.9	65.2	484.5	123.92	312.58
	Total	133	217.59	111.8	193.6	12.2	637.7	134.78	288.55
INVIVO	Female	87	176.21	97.24	151.3	21.5	533.3	99.95	235.45
	Male	46	193.4	99.96	178.8	62.2	417.2	113.62	252.23
	Total	133	182.15	98.15	159.1	21.5	533.3	101.8	237.6
ITK SNAP—Reader 1	Female	87	205.39	100.19	193.33	53.93	550.33	131.1	264.47
	Male	46	213.02	127.06	174.28	39.43	627.57	126.17	284.78
	Total	133	208.03	109.8	185.3	39.43	627.57	129.87	264.6
ITK SNAP—Reader 2	Female	87	205.48	101.14	204	33.78	567	130	263.11
	Male	46	205	115.41	174.5	65	454	111.25	281.5
	Total	133	205.31	105.86	192	33.78	567	120	265

SD—standard deviation, Q1—lower quartile, Q3—upper quartile.

**Table 4:** Correlation between reference, manual (mean measurements of two readers) and automatic (CephX) and semiautomatic (in vivo) measurements.

Method—Variable	ICC	95% CI		Agreement (Koo & Li)
CephX—Total volume	0.945	0.923	0.961	Excellent
CephX—The narrowest area	0.943	0.920	0.959	Excellent
INVIVO—Total volume	0.992	0.989	0.994	Excellent
INVIVO—The narrowest area	0.868	0.819	0.905	Good

ICC—intraclass correlation coefficient; CI—confidence interval.

**Table 5:** Correlation between airway volume and narrowest area.

Variables	Spearman’s Correlation Coefficient	*p*
ITK SNAP—The narrowest area & ITK SNAP—Total volume	0.858	*p* < 0.001

**Table 6:** Repeatability of automated, semiautomated and manual measurements (Reader 1, 20% of cases reanalyzed).

Method—Parameter	ICC	95% CI		Agreement (Koo & Li)
CephX—Total volume	0.998	0.997	0.999	Excellent
CephX—The narrowest area	0.997	0.996	0.998	Excellent
ITK SNAP—Total volume	0.992	0.989	0.995	Excellent
ITK SNAP—The narrowest area	0.957	0.941	0.970	Excellent
INVIVO—Total volume	0.995	0.992	0.996	Excellent
INVIVO—The narrowest area	0.832	0.738	0.890	Good

ICC—intraclass correlation coefficient; CI—confidence interval.

**Table 7:** Average time required for upper airway volume and narrowest area measurements.

Measurement Method	Average Time for Volume (s)	Average Time for Narrowest Area (s)	Total Average Time (s)
Manual (ITK SNAP)	207.6	129	336.6
Semiautomatic (INVIVO)	161.4	Automatically calculated	161.4
Automatic (CephX)	<30	Automatically calculated	<30

Note: Automatic and semiautomatic methods provide the narrowest area measurements simultaneously with volume measurements.

## Data Availability

The data presented in this study are available on request from the corresponding author. The data are not publicly available due to privacy and ethical restrictions.
